# Synthesis, crystal structure and DFT study of carbon­yl[3-(dibenzo[*b*,*d*]thio­phen-4-yl)-6-(pyridin-2-yl-κ*N*)pyridazine-κ*N*^1^]bis­(tri­methyl­phosphane)iron(0)

**DOI:** 10.1107/S2056989025010953

**Published:** 2026-01-01

**Authors:** Ryota Futaki, Noriko Chikaraishi Kasuga, Masakazu Hirotsu

**Affiliations:** a Department of Chemistry, Faculty of Science, Kanagawa University, Kanagawa-ku, Yokohama 221-8686, Japan; Tokyo University of Science, Japan

**Keywords:** crystal structure, iron(0) complex, pyridazine, carbonyl ligand, phosphane ligand, π-back-bonding inter­action

## Abstract

The title compound, [Fe(C_21_H_13_N_3_S)(CO)(PMe_3_)_2_] (**2**), bearing a 3-(dibenzo[*b*,*d*]thio­phen-4-yl)-6-(pyridin-2-yl)pyridazine ligand was obtained by the reaction of [Fe(C_21_H_13_N_3_S)(CO)_3_] (**1**) with [Fe(PMe_3_)_4_]. Crystal structure analysis of **2** revealed that the *N*,*N*-bidentate and three monodentate ligands form a five-coordinate square-pyramidal geometry around Fe, which differs from the trigonal–bipyramidal geometry observed in **1**. The steric and electronic factors were investigated by combining crystal structure and density functional theory (DFT) calculations. Compared with CO, the better σ donor properties of PMe_3_ induce the square-pyramidal geometry of **2**, and a significant π-back-bonding inter­action was confirmed between Fe and the pyridazine moiety.

## Abstract 2

The title compound [Fe(C_21_H_13_N_3_S)(CO)(PMe_3_)_2_] exhibits a square-pyramidal geometry, distinct from the trigonal–bipyramidal geometry in [Fe(C_21_H_13_N_3_S)(CO)_3_], due to the σ-donating properties of the two tri­methyl­phosphane ligands. An increase in π-back-donation from the electron-rich iron center to the pyridazine moiety was also confirmed.

## 1. Chemical context

The utilization of earth-abundant transition-metal catalysts has become increasingly important, and iron-catalyzed reactions are attractive research topics (Bolm *et al.*, 2004 ▸; Bauer & Knölker, 2015 ▸; Takeda *et al.*, 2017 ▸; Ekspong *et al.*, 2021 ▸; Gao *et al.*, 2022 ▸; Sila *et al.*, 2024 ▸). Low-valent iron complexes have been studied intensively because their unsaturated coordination sites are highly active and play a pivotal role in catalytic reactions (Toya *et al.*, 2017 ▸; Takeshita *et al.*, 2018 ▸; Nakajima *et al.*, 2025 ▸; Zhang *et al.*, 2025 ▸; Wang *et al.*, 2025 ▸). Iron carbonyls are attractive catalysts and precatalysts for fundamental organic and inorganic reactions (Schroeder & Wrighton, 1976 ▸; Petricci *et al.*, 2018 ▸; Hirayama *et al.*, 2025 ▸; Torrent *et al.*, 2000 ▸). For example, [Fe(CO)_5_] catalyzes the water**–**gas shift reaction and isomerization of olefins (Schroeder & Wrighton, 1976 ▸), and [Fe_2_(CO)_9_] and [Fe_3_(CO)_12_] are catalyst precursors for the reductive amination of aldehydes (Petricci *et al.*, 2018 ▸; Hirayama *et al.*, 2025 ▸). Furthermore, various diiron carbonyl complexes with thiol­ate ligands have been synthesized from [Fe(CO)_5_] and are promising candidates for the active-site model of [FeFe]-hydrogenases (Tard & Pickett, 2009 ▸).

We previously reported diiron hydrogenase mimics bearing thiol­ate ligands, which were synthesized by the photoreactions of [Fe(CO)_5_] with dibenzo­thio­phene derivatives containing Schiff base or pyridine moieties *via* C—S bond cleavage (Hirotsu *et al.*, 2012 ▸, 2014 ▸; Nakae *et al.*, 2015 ▸). However, the corresponding reaction with 3-(dibenzo[*b*,*d*]thio­phen-4-yl)-6-(pyridin-2-yl)pyridazine (dbtpdzpy) gave the iron(0) carbonyl complex [Fe(dbtpdzpy)(CO)_3_] (**1**), in which the dbtpdzpy ligand is bound to Fe in an *N*,*N*-bidentate fashion, forming a five-membered chelate ring (Futaki *et al.*, 2025 ▸). We carried out the reaction of **1** with the reactive iron(0) complex [Fe(PMe_3_)_4_] in THF, expecting the remaining N atom to act as a directing group for C—S bond activation. However, the N,S-coordination site remained intact, and the five-coordinate iron(0) complex [Fe(dbtpdzpy)(CO)(PMe_3_)_2_] (**2**) was obtained, in which the two carbonyl ligands in **1** are replaced by PMe_3_ dissociated from [Fe(PMe_3_)_4_]. The reaction of **1** with PMe_3_ gives dbtpdzpy and carbonyl tri­methyl­phosphane iron(0) complexes such as [Fe(CO)_3_(PMe_3_)_2_]; therefore, this ligand scrambling reaction provides a suitable route for the preparation of **2**. The crystal structure of the bis­(tri­methyl­phosphane) iron(0) complex **2** showed geometrical changes around the Fe from **1**, including a significant shortening of the Fe—N(pyridazine) bonds, which is discussed in terms of the steric and electronic effects of PMe_3_.
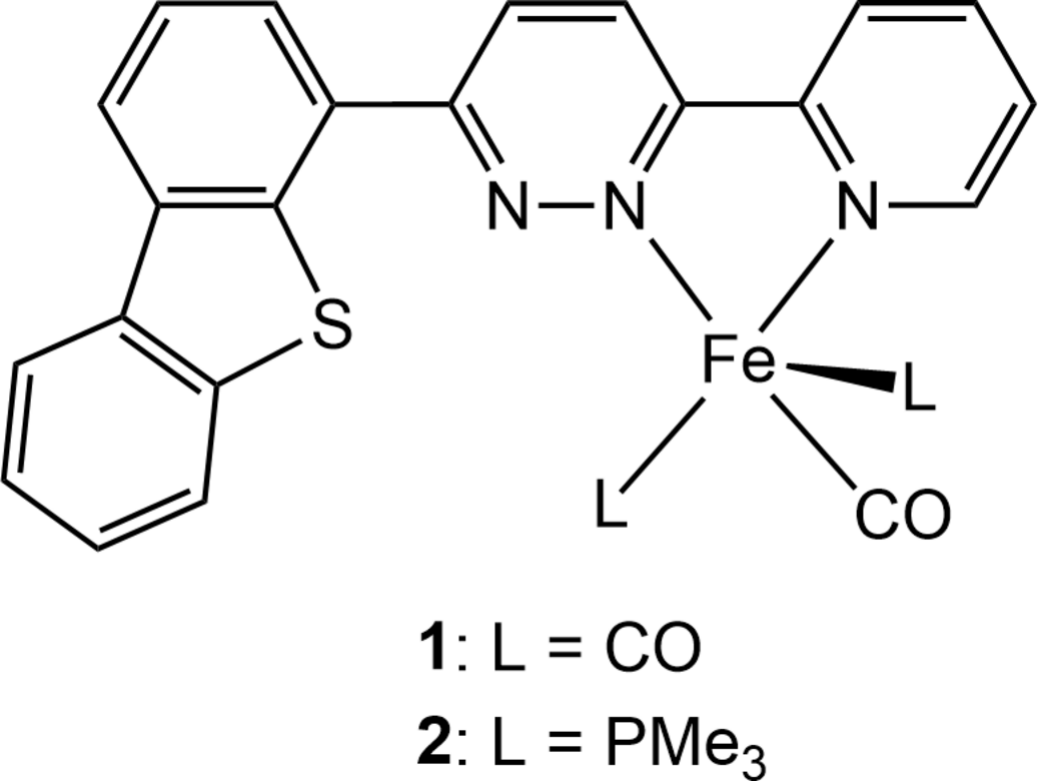


## 2. Structural commentary

**Figure 1:**
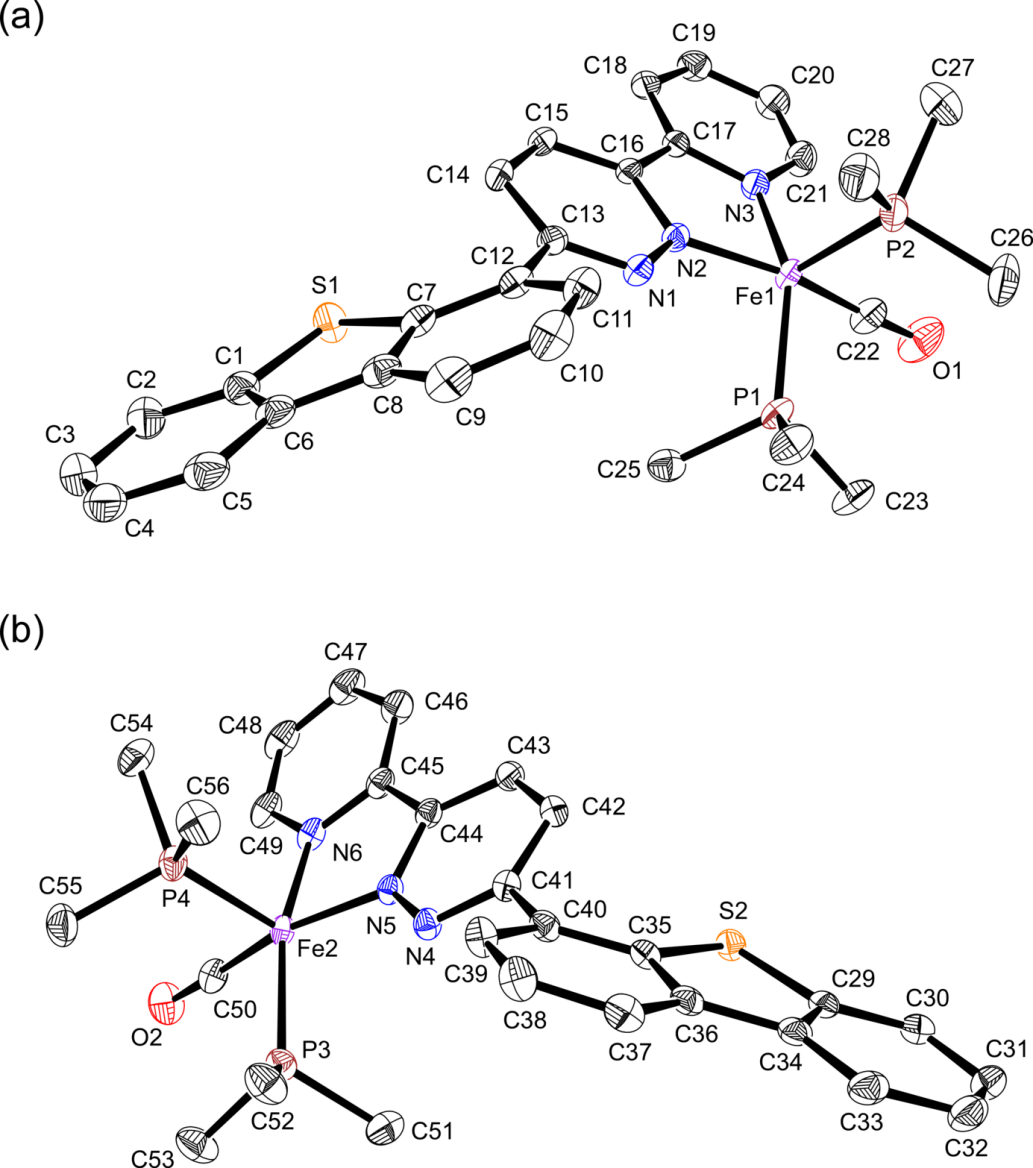
Perspective view of mol­ecules (*a*) 2A and (*b*) 2B in **2** with displacement ellipsoids at the 50% probability level. Hydrogen atoms are omitted for clarity.

**Figure 2:**
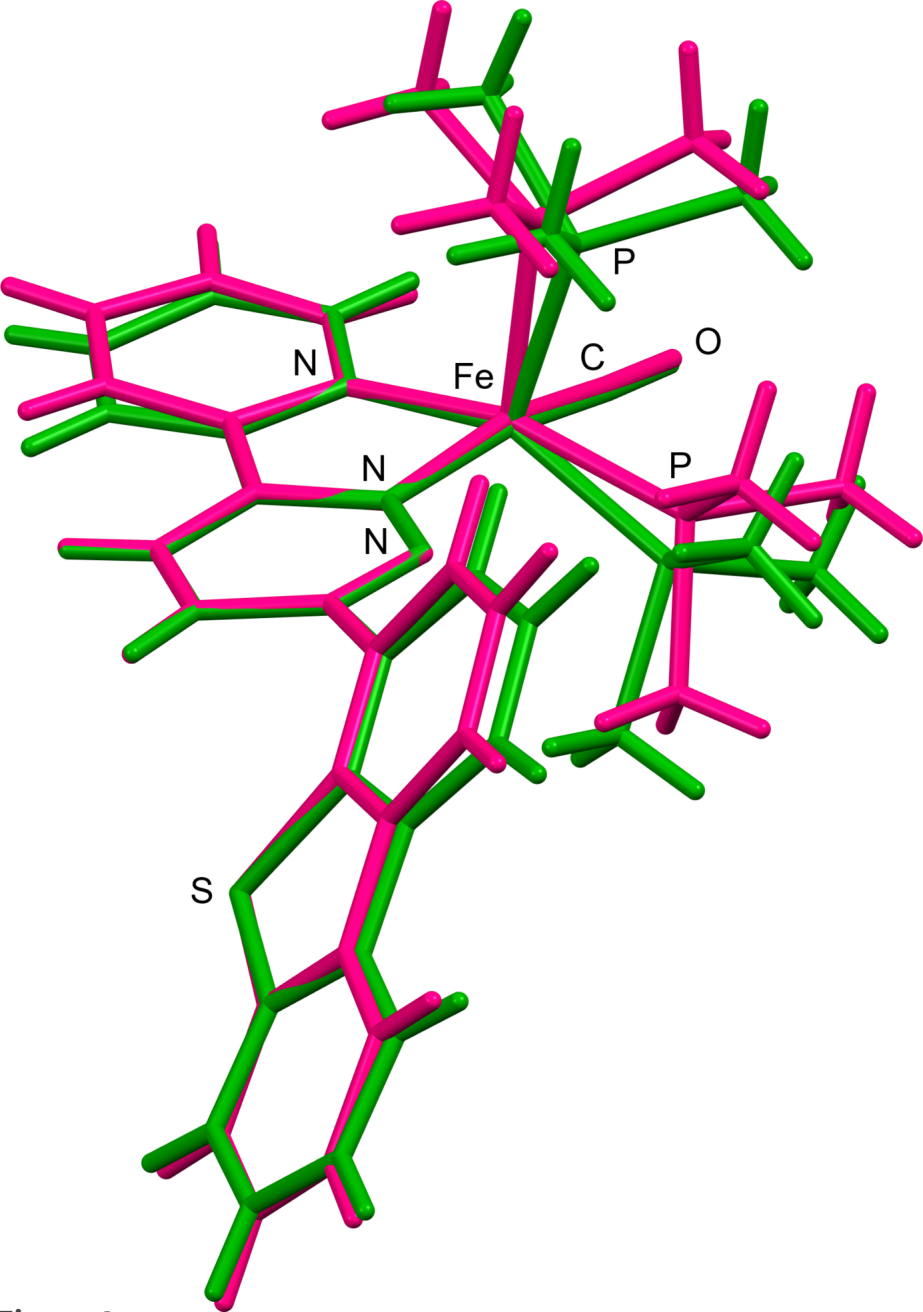
Overlay of mol­ecules 2A (green, symmetry operation: *x*, *y*, *z*) and 2B (magenta, symmetry operation: *x* − 1, −*y* + 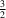, *z* − 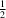).

Complex **2** crystallizes in the centrosymmetric space group *P*2_1_/*c* (No. 14) with two independent [Fe(dbtpdzpy)(CO)(PMe_3_)_2_] mol­ecules, 2A and 2B, in the asymmetric unit (Fig. 1 ▸). Selected bond distances and angles are listed in Table 1 ▸. The five-coordinate iron center is chelated by the *N*,*N*-bidentate ligand dbtpdzpy, and the carbonyl ligand is located *trans* to the pyridazine N atom. The remaining sites are occupied by two PMe_3_ ligands, and the dibenzo[*b*,*d*]thio­phen-4-yl group is oriented away from the PMe_3_ ligands. This contrasts with the near planar arrangement of the aromatic rings observed in **1**. Both mol­ecules 2A and 2B have a distorted square-pyramidal coordination geometry, and one of the two PMe_3_ ligands occupies the apical position of the pyramid. The structural index parameter τ is 0.18 for 2A and 0.023 for 2B, suggesting a larger distortion of 2A from the ideal square-pyramidal geometry (Addison *et al.*, 1984 ▸). The differences are visualized by overlaying the mol­ecular structures of 2A and 2B (symmetry operation: *x* − 1, −*y* + 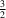, *z* − 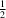), where the central pyridazine ring is superposed (Fig. 2 ▸). Significant differences were detected in the two N(pyridine)—Fe—P angles and the inter­planar angles between the dibenzo­thio­phene/pyridazine/pyridine moieties.

In the crystal structure of **2**, the two PMe_3_ ligands occupy the less crowded apical and basal positions. The stronger σ donor properties of PMe_3_ relative to CO make the Fe center of **2** more electron-rich than that of **1**. This affects the bond lengths around Fe due to enhanced π-back-bonding inter­actions. The Fe—C(carbon­yl) bond lengths of **2** [1.7426 (17), 1.7371 (16) Å] are shorter than those of **1** [1.771 (2), 1.801 (2), 1.7774 (18) Å]. This is consistent with a large red shift of the CO stretching frequency for **2** (1844 cm^−1^) compared to **1** (1984 cm^−1^, 1925 cm^−1^, 1894 cm^−1^). Furthermore, the Fe—N(pyridazine) bond lengths of **2** [1.9054 (12), 1.9117 (12) Å] are approximately 0.04 Å shorter than that of **1** [1.9487 (14) Å], whereas the N—N(coordinated) [1.3736 (17), 1.3826 (16) Å] and N(coordinated)—C [1.3954 (18), 1.3964 (18) Å] bond lengths in the pyridazine ring of **2** are longer than those of **1** [N—N(coordinated), 1.3600 (18) Å; N(coordinated)—C, 1.363 (2) Å], respectively. These findings are consistent with the moderate π acceptor character of pyridazine.

## 3. Density functional theory calculations

**Figure 3:**
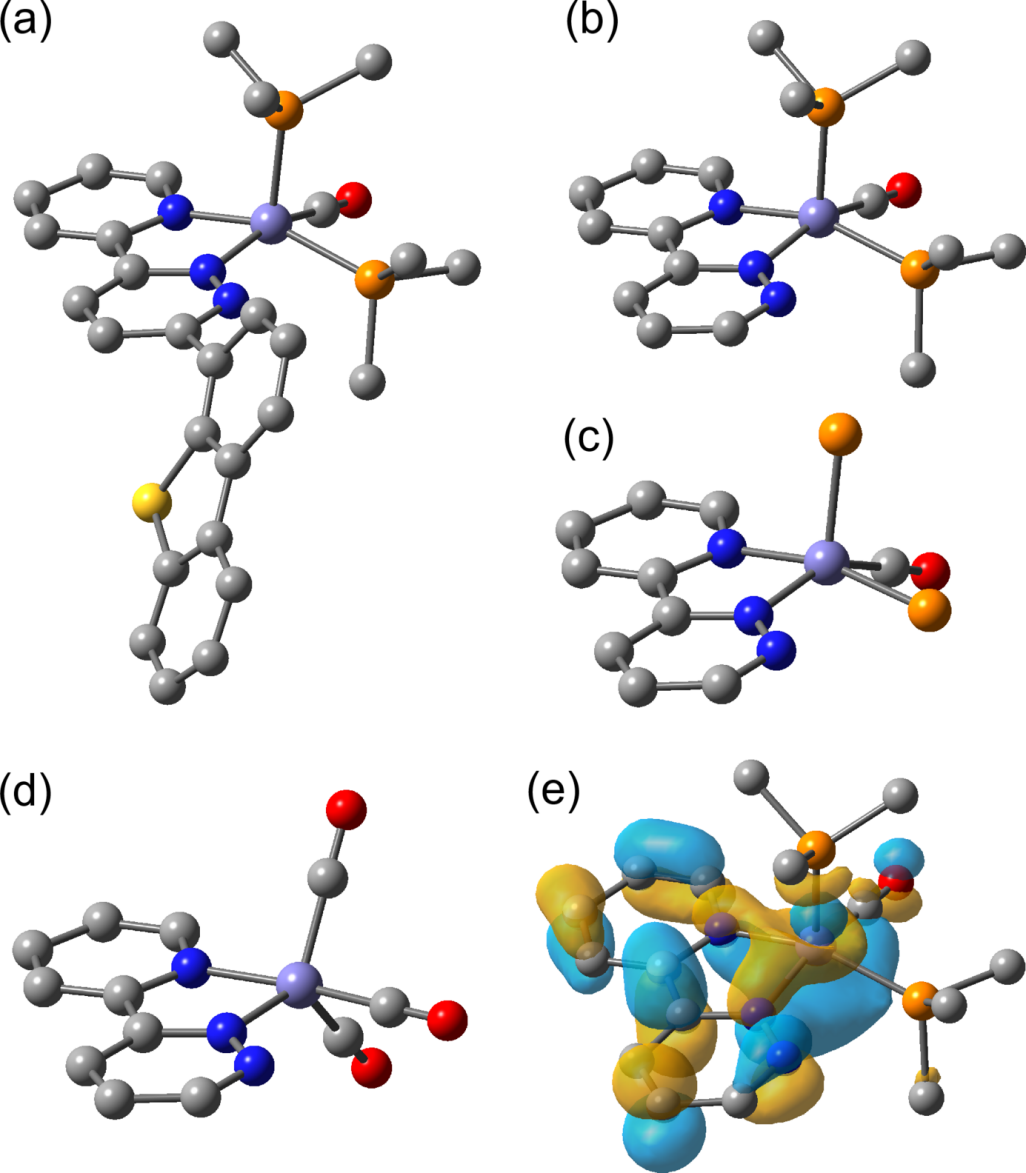
Optimized structures of (*a*) **2**, (*b*) [Fe(pypdz)(CO)(PMe_3_)_2_] (**3**), (*c*) [Fe(pypdz)(CO)(PH_3_)_2_] (**4**), and (*d*) [Fe(pypdz)(CO)_3_] (**5**). (*e*) HOMO of **3** (isovalue = 0.03 Bohr^−3/2^). Hydrogen atoms are omitted for clarity.

To analyze τ value differences, the structures of **2** and related complexes were optimized by density functional theory (DFT) calculations at the B3LYP/6-311+G(d,p) level. All DFT calculations in this study were carried out using the *Gaussian 16* program (Frisch *et al.*, 2016 ▸), and the results were visualized using the *GaussView 6.0* software (Dennington *et al.*, 2016 ▸). The resulting structural indices τ and selected geometric parameters are summarized in Table 2 ▸. The crystal structures of 2A and 2B were used as the initial models for the calculations, which yielded identical results (Fig. 3 ▸*a*). The τ value of 0.017 for the optimized structure is close to that of 2B. To exclude the influence of the dibenzo­thio­phene moiety, the structure of [Fe(pypdz)(CO)(PMe_3_)_2_] (**3**) (pypdz = 3-(pyridin-2-yl)pyridazine) was also optimized (Fig. 3 ▸*b*), and a τ value of 0.045 was obtained. These results suggest that the geometry around the Fe atom of 2A is more strongly influenced by the crystal packing effects, as mentioned later.

The square-pyramidal geometry of **2** differs from the trigonal–bipyramidal geometry of **1** (τ = 0.65). The pyridazine moiety in **1** occupies an equatorial position, which is likely due to its better π acceptor properties than those of pyridine. The bite angles of the *N*,*N*-chelate in 2A [80.70 (5)°] and 2B [80.86 (5)°] are comparable to that in **1** [80.27 (6)°)]. To conform the preference for the square-pyramidal geometry in **2** while excluding the packing and substituent effects, the structure of the analogous iron complex with the less bulky PH_3_ ligands, [Fe(pypdz)(CO)(PH_3_)_2_] (**4**), was optimized by DFT calculations (Fig. 3 ▸*c*). The τ value of 0.29 suggests that complex **4** has a distorted square-pyramidal geometry. Furthermore, the optimized structure of [Fe(pypdz)(CO)_3_] (**5**) (Fig. 3 ▸*d*, τ = 0.76) is similar to that of the bi­pyridine complex [Fe(bpy)(CO)_3_] (bpy = 2,2′-bi­pyridine) with a trigonal–bipyramidal geometry: τ = 0.83 (Calderazzo *et al.*, 2002 ▸), 0.81 (DelaVarga *et al.*, 2003 ▸). Therefore, the geometrical change between **1** and **2** is mainly attributable to the electronic effect of the PMe_3_ ligand, which is a better σ-donor and poorer π-acceptor ligand than CO. Fig. 3 ▸*e* shows the HOMO of the bis­(tri­methyl­phosphane) complex **2**, which is located over the pypdz ligand and the iron center. This mol­ecular orbital is a combination of the metal d orbital and the LUMO of pypdz, showing the π-back-donation from Fe to pypdz. Natural population analysis of **3** (natural charge (*e*): Fe, −1.145; pyridine, −0.042; pyridazine, −0.241) and **5** (natural charge (*e*): Fe, −1.240; pyridine, 0.165; pyridazine, 0.016) also revealed that the pyridazine moiety in the PMe_3_ complex **3** effectively accepts electron density from Fe: the difference in natural charge between **3** and **5** is 0.207*e* in the pyridine moiety and 0.257*e* in the pyridazine moiety.

## 4. Supra­molecular features

**Figure 4:**
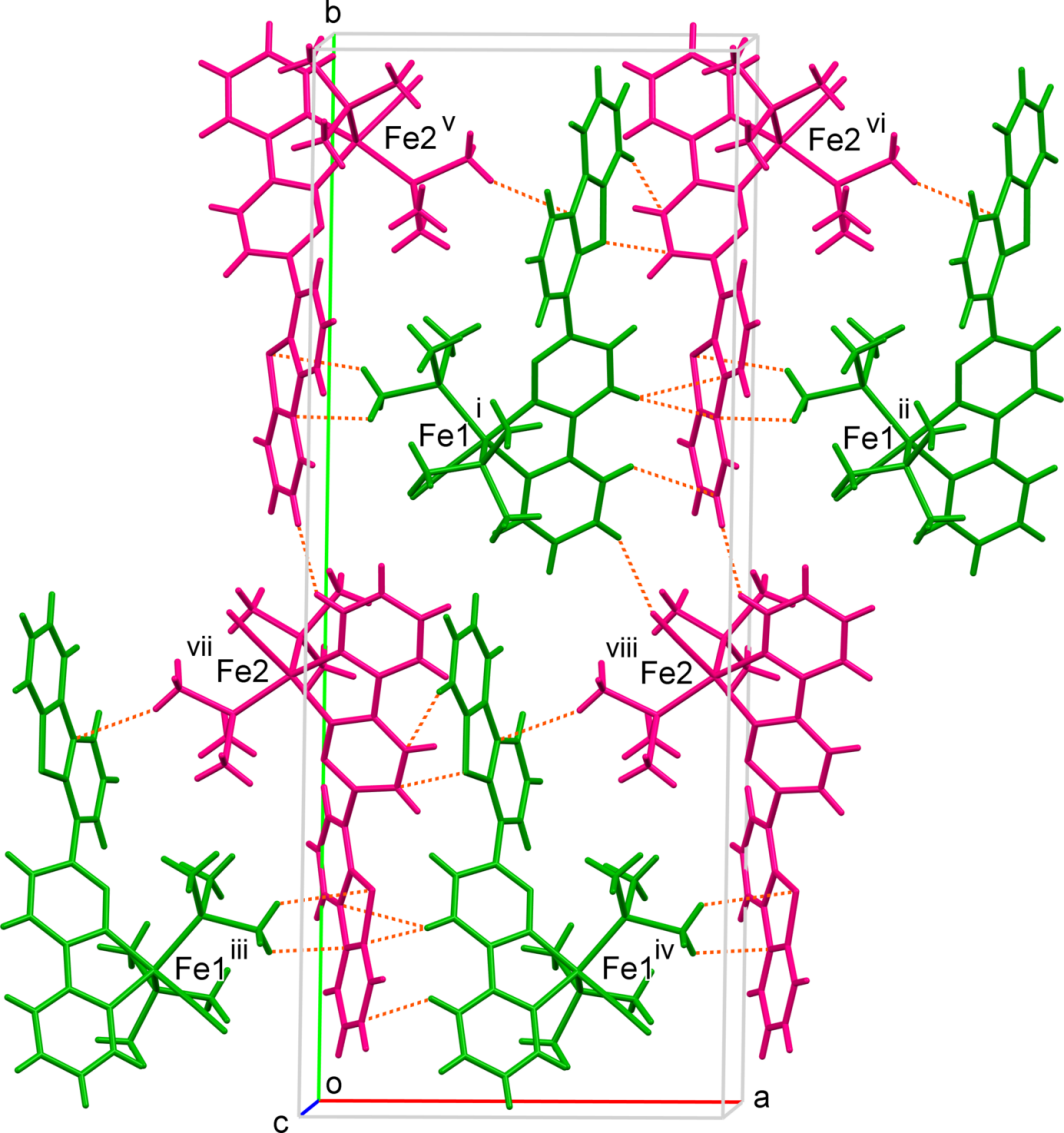
Partial view of the crystal packing of **2**, showing a pair of 2A (green) and 2B (magenta) mol­ecules. Short contacts are shown as dashed lines. [Symmetry codes: (i) *x*, *y*, *z*; (ii) *x* + 1, *y*, *z*; (iii) −*x*, *y* − 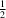, −*z* + 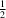; (iv) −*x* + 1, *y* − 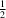, −*z* + 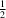; (v) *x* − 1, −*y* + 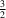, *z* − 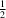; (vi) *x*, −*y* + 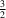, *z* − 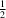; (vii) −*x* + 1, −*y* + 1, −*z* + 1; (viii) −*x* + 2, −*y* + 1, −*z* + 1.]

Mol­ecules 2A (symmetry operation: *x*, *y*, *z*) and 2B (symmetry operation: *x* − 1, −*y* + 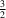, *z* − 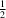) are related by a pseudo *C*_2_ axis along the *c* axis in the crystal, and four pairs located on the *ab* plane are shown in Fig. 4 ▸. The PMe_3_ ligand at the basal position is located near the dibenzo­thio­phene moiety of the paired mol­ecule. The basal PMe_3_ ligands are fitted into a cavity consisting of aromatic ligands. Some short contacts are found between 2A and 2B, mainly along the *a* axis, which are attributed to C—H⋯π inter­actions: *e.g*., H15⋯C34(*x*, −*y* + 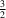, *z* − 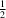), H2⋯C43(*x*, −*y* + 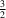, *z* − 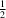) C8⋯C53*B*(*x* − 1, −*y* + 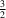, *z* − 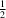) (Table 1 ▸).

**Figure 5:**
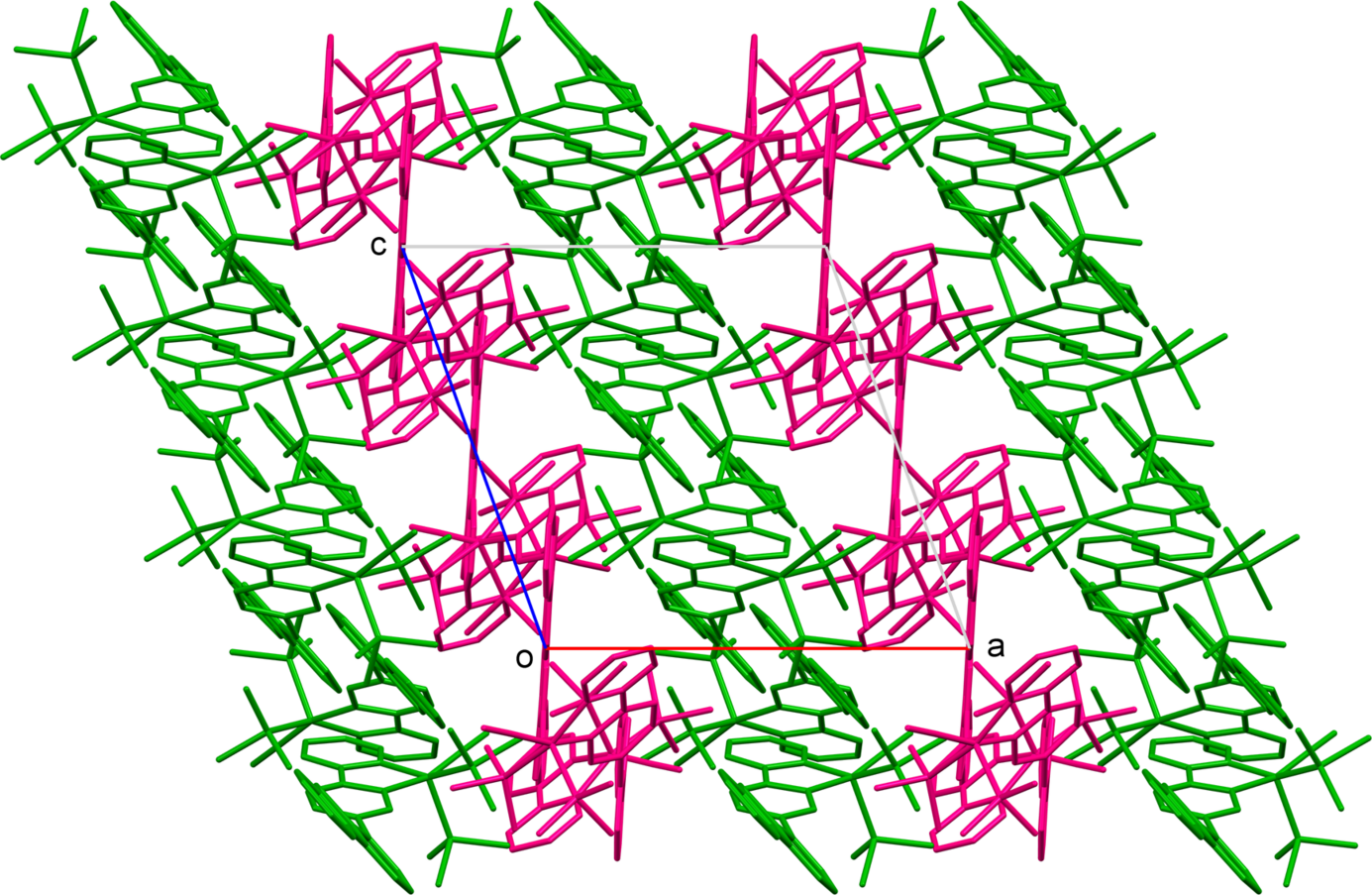
Crystal packing diagram of **2** along the *b* axis, showing the layers of 2A (green) and 2B (magenta).

**Figure 6:**
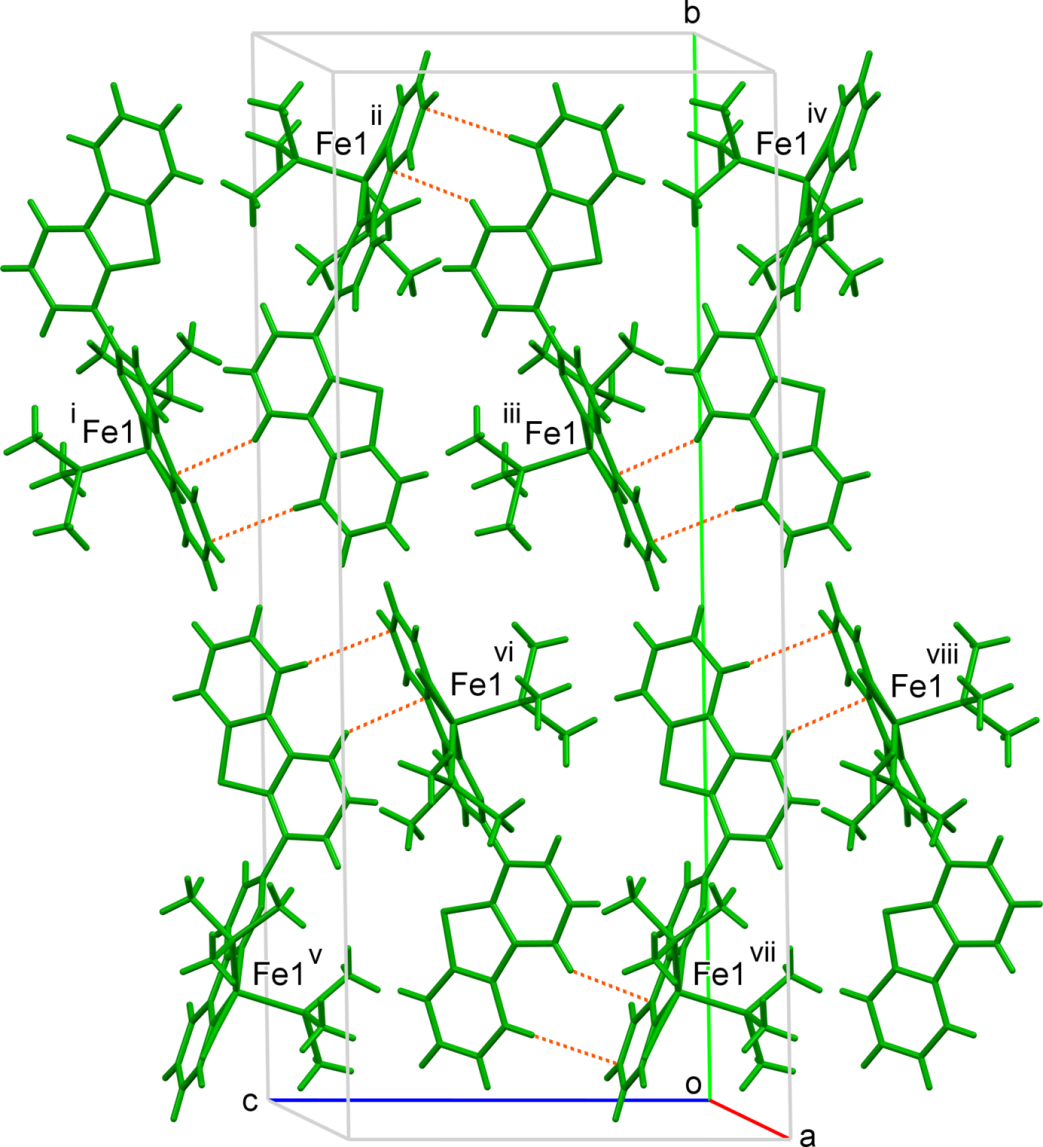
Partial view of the crystal packing of **2**, showing the layers of 2A. Short contacts are shown as dashed lines. [Symmetry codes: (i) *x*, *y*, *z* + 1; (ii) *x*, −*y* + 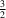, *z* + 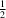; (iii) *x*, *y*, *z*; (iv) *x*, −*y* + 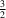, *z* − 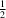; (v) −*x* + 1, *y* − 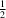, −*z* + 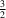 (vi) −*x* + 1, −*y* + 1, −*z* + 1 (vii) −*x* + 1, *y* − 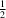, −*z* + 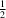 (viii) −*x* + 1, −*y* + 1, −*z*.]

Fig. 5 ▸ shows the crystal packing of **2** along the *b* axis. The layers of 2A and 2B are parallel to the *bc* plane and stacked alternately along the *a* axis. As shown in Fig. 6 ▸, short contacts due to C—H⋯π inter­actions are observed only in the 2A layer: H9⋯C17(*x*, −*y* + 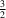, *z* + 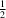), H5⋯C19(*x*, −*y* + 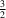, *z* + 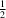) (Table 1 ▸). The crystal packing affects the inter­planar angles of the dibenzo­thio­phene and pyridazine moieties of 2A. This induces a deviation in the N(pyridine)—Fe—P angles through steric inter­actions. Therefore, 2A is more distorted from the ideal square-pyramidal geometry than 2B.

## 5. Database survey

Various transition-metal complexes containing a pyridyl­pyridazine unit in the ligands have been structurally characterized by X-ray crystallography: Fe (Futaki *et al.*, 2025 ▸; Guo *et al.*, 2021 ▸), Ru (De Munno *et al.*, 1988 ▸; Xu *et al.*, 2009 ▸), Pt (McCready & Puddephatt, 2015 ▸), Co, Ni, Zn (Savjani *et al.*, 2015 ▸), Re (Sangilipandi *et al.*, 2015 ▸; Mosberger *et al.*, 2019 ▸; Schnierle *et al.*, 2022 ▸). However, structural reports on iron complexes are rare (Futaki *et al.*, 2025 ▸; Guo *et al.*, 2021 ▸).

## 6. Synthesis and crystallization

A suspension of complex **1** (0.058 g, 0.12 mmol) and [Fe(PMe_3_)_4_] (0.016 g, 0.11 mmol) in tetrahydrofuran (THF) (10 mL) was stirred at room temperature under a nitro­gen atmosphere for 1 d. The resulting deep brown solution was concentrated to dryness under reduced pressure to afford a red–brown solid. The solid was dissolved in toluene, and hexane was layered on top of the solution. The mixture was cooled to 253 K, and the precipitated red–brown solid was removed by deca­ntation. The resulting blue–green solution was stored at 253 K to yield **2** as red–brown crystals (3.6 mg, 5%). The low yield is probably due to the high solubility of **2** in toluene and hexane. ^1^H NMR (600 MHz, C_6_D_6_): δ 9.83 (*d*, *J* = 6.2 Hz, 1H), 7.94 (*m*, 2H), 7.89 (*d*, *J* = 8.0 Hz, 1H), 7.55 (*dd*, *J* = 8.0, 0.4 Hz, 1H), 7.44 (*td*, *J* = 7.6, 0.4 Hz, 1H), 7.32 (*m*, 1H), 7.26 (*dd*, *J* = 8.6, 0.4 Hz, 1H), 7.21 (*m*, 1H), 7.15 (*m*, 1H), 6.97 (*td*, *J* = 8.6, 1.4 Hz, 1H), 6.80 (*m*, 1H), 6.52 (*td*, *J* = 6.6, 1.0 Hz, 1H), 1.05 (*d*, *J* = 7.6 Hz, 18H). ^31^P{^1^H} NMR (243 MHz, C_6_D_6_): δ 22.7. *ν*_CO_/cm^−1^ (KBr): 1844. Analysis calculated for C_28_H_31_FeN_3_OP_2_S·0.9H_2_O: C; 56.84, H; 5.59, N; 7.10. Found: C, 56.52; H, 5.27; N, 7.59.

## 7. Refinement

Crystal data, data collection and structure refinement details are summarized in Table 3 ▸. All non-hydrogen atoms were refined anisotropically. Hydrogen atoms were placed in calculated positions with C—H(aromatic) = 0.95 Å and C—H(meth­yl) = 0.98 Å, and refined using a riding model with *U*_iso_(H) = 1.2*U*_eq_(C) and 1.5*U*_eq_(C), respectively.

## Supplementary Material

Crystal structure: contains datablock(s) I. DOI: 10.1107/S2056989025010953/jp2020sup1.cif

Structure factors: contains datablock(s) I. DOI: 10.1107/S2056989025010953/jp2020Isup2.hkl

CCDC reference: 2513314

Additional supporting information:  crystallographic information; 3D view; checkCIF report

## Figures and Tables

**Table 1:** Selected geometric parameters (Å, °)

Fe1—P1	2.2144 (4)	Fe2—P3	2.2087 (4)
Fe1—P2	2.1847 (5)	Fe2—P4	2.1862 (4)
Fe1—N2	1.9054 (12)	Fe2—N5	1.9117 (12)
Fe1—N3	1.9464 (13)	Fe2—N6	1.9434 (13)
Fe1—C22	1.7426 (17)	Fe2—C50	1.7371 (16)
			
H9⋯C17^i^	2.676	H23*C*⋯C34^iii^	2.824
H15⋯C34^ii^	2.704	H25*C*⋯C52^v^	2.834
H2⋯C43^ii^	2.730	H49^vi^⋯H32^iii^	2.336
C8⋯H53*B*^iii^	2.755	C6⋯H30	2.841
H15⋯C36^ii^	2.770	H23*B*⋯S2^iii^	2.943
S1⋯C42^ii^	3.389	H5⋯C19^i^	2.843
O2⋯H48^iv^	2.616	H19⋯O2^iv^	2.664
H18⋯C32^ii^	2.798		
			
P2—Fe1—P1	99.457 (18)	P4—Fe2—P3	98.499 (17)
N2—Fe1—P1	91.04 (4)	N5—Fe2—P3	94.01 (4)
N2—Fe1—P2	102.65 (4)	N5—Fe2—P4	102.48 (4)
N3—Fe1—P1	153.19 (4)	N6—Fe2—P3	163.19 (4)
C22—Fe1—N2	163.93 (7)	C50—Fe2—N5	161.83 (6)

Symmetry codes: (i) 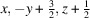; (ii) 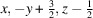; (iii) 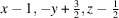; (iv) 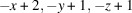; (v) 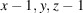; (vi) 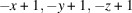.

**Table 2:** Structural indices (τ) and geometric parameters (Å, °) for experimental (**1**, 2A in **2**, 2B in **2**) and computational (**2**, **3**, **4**, **5**) results

	**1** ^*a*^	2A^*b*^	2B^*b*^	**2** ^*c*^	**3** ^*c*^	**4** ^*c*^	**5** ^*c*^
τ	0.649	0.179	0.023	0.017	0.045	0.293	0.759
Fe—N(pyridazine)	1.9487 (14)	1.9054 (12)	1.9117 (12)	1.914	1.909	1.892	1.927
Fe—N(pyridine)	1.9622 (14)	1.9464 (13)	1.9434 (13)	1.971	1.971	1.966	1.987
N—Fe—N	80.27 (6)	80.70 (5)	80.86 (5)	80.75	80.65	80.86	80.31
Inter­planar angle^d^	3.94 (7)	46.79 (4)	52.56 (4)	55.79	–	–	–
Inter­planar angle^e^	4.02 (9)	3.03 (8)	9.63 (8)	0.57	1.18	0.35	0

Notes: (*a*) Futaki *et al.* (2025 ▸); (*b*) experimental data; (*c*) data from DFT calculations; (*d*) inter­planar angles were calculated as the dihedral angle between the least-squares planes of the dibenzo­thio­phene and pyridazine moieties; (*e*) inter­planar angles were calculated as the dihedral angle between the least-squares planes of the pyridazine and pyridine moieties.

**Table 3:** Experimental details

Crystal data	
Chemical formula	[Fe(C_21_H_13_N_3_S)(C_3_H_9_P)_2_(CO)]
*M*_r_	575.41
Crystal system, space group	Monoclinic, *P*2_1_/*c*
Temperature (K)	90
*a*, *b*, *c* (Å)	13.4551 (3), 32.3786 (7), 13.5356 (3)
β (°)	109.700 (3)
*V* (Å^3^)	5551.7 (2)
*Z*	8
Radiation type	Mo *K*α
μ (mm^−1^)	0.76
Crystal size (mm)	0.24 × 0.21 × 0.08

Data collection	
Diffractometer	ROD, Synergy Custom system, HyPix
Absorption correction	Multi-scan (*CrysAlis PRO*; Rigaku OD, 2024 ▸)
*T*_min_, *T*_max_	0.878, 1.000
No. of measured, independent and observed [*I* > 2σ(*I*)] reflections	62645, 14433, 12163
*R*_int_	0.033
(sin θ/λ)_max_ (Å^−1^)	0.725

Refinement	
*R*[*F*^2^ > 2σ(*F*^2^)], *wR*(*F*^2^), *S*	0.031, 0.083, 1.04
No. of reflections	14433
No. of parameters	661
H-atom treatment	H-atom parameters constrained
Δρ_max_, Δρ_min_ (e Å^−3^)	0.52, −0.39

Computer programs: *CrysAlis PRO* (Rigaku OD, 2024 ▸), *SHELXT2018/2* (Sheldrick, 2015*a* ▸), *SHELXL2019/3* (Sheldrick, 2015*b* ▸) and *OLEX2* (Dolomanov *et al.*, 2009 ▸).

## supplementary crystallographic information

### Carbonyl[3-(dibenzo[b,d]thiophen-4-yl)-6-(pyridin-2-yl-κN)pyridazine-κN1]bis(trimethylphosphane)iron(0) Crystal data

**Table:**

[Fe(C_21_H_13_N_3_S)(C_3_H_9_P)_2_(CO)]	*F*(000) = 2400
*M**_r_* = 575.41	*D*_x_ = 1.377 Mg m^−^^3^
Monoclinic, *P*2_1_/*c*	Mo *K*α radiation, λ = 0.71073 Å
*a* = 13.4551 (3) Å	Cell parameters from 32963 reflections
*b* = 32.3786 (7) Å	θ = 3.0–31.0°
*c* = 13.5356 (3) Å	µ = 0.76 mm^−^^1^
β = 109.700 (3)°	*T* = 90 K
*V* = 5551.7 (2) Å^3^	Block, dark red
*Z* = 8	0.24 × 0.21 × 0.08 mm

### Carbonyl[3-(dibenzo[b,d]thiophen-4-yl)-6-(pyridin-2-yl-κN)pyridazine-κN1]bis(trimethylphosphane)iron(0) Data collection

**Table:**

ROD, Synergy Custom system, HyPix diffractometer	14433 independent reflections
Radiation source: Rotating-anode X-ray tube, Rigaku (Mo) X-ray Source	12163 reflections with *I* > 2σ(*I*)
Mirror monochromator	*R*_int_ = 0.033
Detector resolution: 10.0000 pixels mm^-1^	θ_max_ = 31.0°, θ_min_ = 3.0°
ω scans	*h* = −17→18
Absorption correction: multi-scan (CrysAlisPro; Rigaku OD, 2024)	*k* = −43→45
*T*_min_ = 0.878, *T*_max_ = 1.000	*l* = −19→18
62645 measured reflections	

### Carbonyl[3-(dibenzo[b,d]thiophen-4-yl)-6-(pyridin-2-yl-κN)pyridazine-κN1]bis(trimethylphosphane)iron(0) Refinement

**Table:**

Refinement on *F*^2^	Primary atom site location: dual
Least-squares matrix: full	Hydrogen site location: inferred from neighbouring sites
*R*[*F*^2^ > 2σ(*F*^2^)] = 0.031	H-atom parameters constrained
*wR*(*F*^2^) = 0.083	*w* = 1/[σ^2^(*F*_o_^2^) + (0.0401*P*)^2^ + 2.4796*P*] where *P* = (*F*_o_^2^ + 2*F*_c_^2^)/3
*S* = 1.04	(Δ/σ)_max_ = 0.002
14433 reflections	Δρ_max_ = 0.52 e Å^−^^3^
661 parameters	Δρ_min_ = −0.38 e Å^−^^3^
0 restraints	

### Carbonyl[3-(dibenzo[b,d]thiophen-4-yl)-6-(pyridin-2-yl-κN)pyridazine-κN1]bis(trimethylphosphane)iron(0) Special details

**Table:**

Geometry. All esds (except the esd in the dihedral angle between two l.s. planes) are estimated using the full covariance matrix. The cell esds are taken into account individually in the estimation of esds in distances, angles and torsion angles; correlations between esds in cell parameters are only used when they are defined by crystal symmetry. An approximate (isotropic) treatment of cell esds is used for estimating esds involving l.s. planes.

### Carbonyl[3-(dibenzo[b,d]thiophen-4-yl)-6-(pyridin-2-yl-κN)pyridazine-κN1]bis(trimethylphosphane)iron(0) Fractional atomic coordinates and isotropic or equivalent isotropic displacement parameters (Å^2^)

**Table:**

	*x*	*y*	*z*	*U*_iso_*/*U*_eq_	
Fe1	0.38565 (2)	0.62411 (2)	0.32029 (2)	0.01969 (5)	
S1	0.65732 (3)	0.81061 (2)	0.34172 (3)	0.02582 (8)	
P1	0.27314 (3)	0.67629 (2)	0.29259 (3)	0.02223 (8)	
P2	0.38516 (3)	0.60425 (2)	0.47438 (3)	0.02675 (9)	
O1	0.21163 (11)	0.56935 (5)	0.21116 (13)	0.0481 (4)	
N1	0.50321 (9)	0.69917 (4)	0.40088 (10)	0.0198 (2)	
N2	0.50212 (9)	0.66130 (4)	0.35479 (10)	0.0183 (2)	
N3	0.48680 (10)	0.58951 (4)	0.28443 (10)	0.0217 (3)	
C1	0.65719 (12)	0.86409 (5)	0.36221 (13)	0.0246 (3)	
C2	0.69065 (14)	0.89446 (5)	0.30716 (15)	0.0330 (4)	
H2	0.717215	0.887173	0.252789	0.040*	
C3	0.68421 (14)	0.93547 (6)	0.33368 (17)	0.0368 (4)	
H3	0.705747	0.956544	0.296416	0.044*	
C4	0.64648 (13)	0.94620 (5)	0.41441 (16)	0.0341 (4)	
H4	0.643307	0.974455	0.432018	0.041*	
C5	0.61366 (13)	0.91610 (5)	0.46902 (14)	0.0298 (4)	
H5	0.587732	0.923626	0.523684	0.036*	
C6	0.61888 (12)	0.87442 (5)	0.44327 (13)	0.0239 (3)	
C7	0.60539 (11)	0.80132 (5)	0.44243 (12)	0.0206 (3)	
C8	0.58965 (11)	0.83825 (5)	0.49030 (12)	0.0223 (3)	
C9	0.54778 (13)	0.83641 (5)	0.57167 (13)	0.0285 (3)	
H9	0.536400	0.861084	0.604326	0.034*	
C10	0.52310 (14)	0.79876 (6)	0.60427 (13)	0.0306 (4)	
H10	0.496256	0.797487	0.660745	0.037*	
C11	0.53711 (12)	0.76234 (5)	0.55518 (12)	0.0256 (3)	
H11	0.518623	0.736683	0.578313	0.031*	
C12	0.57760 (11)	0.76281 (5)	0.47301 (11)	0.0205 (3)	
C13	0.58684 (11)	0.72335 (4)	0.42035 (11)	0.0192 (3)	
C14	0.67901 (11)	0.71251 (5)	0.39751 (12)	0.0221 (3)	
H14	0.736322	0.731176	0.409316	0.026*	
C15	0.68116 (11)	0.67382 (5)	0.35766 (12)	0.0216 (3)	
H15	0.742054	0.664646	0.343480	0.026*	
C16	0.59346 (11)	0.64774 (4)	0.33777 (11)	0.0180 (3)	
C17	0.58394 (11)	0.60719 (5)	0.29734 (11)	0.0194 (3)	
C18	0.66233 (12)	0.58596 (5)	0.26952 (13)	0.0239 (3)	
H18	0.727201	0.599297	0.276343	0.029*	
C19	0.64543 (13)	0.54640 (5)	0.23288 (13)	0.0277 (3)	
H19	0.697584	0.532025	0.213456	0.033*	
C20	0.54883 (14)	0.52740 (5)	0.22459 (14)	0.0302 (3)	
H20	0.535820	0.499579	0.201572	0.036*	
C21	0.47386 (13)	0.54929 (5)	0.24990 (14)	0.0290 (3)	
H21	0.409064	0.535920	0.243231	0.035*	
C22	0.28045 (13)	0.59203 (5)	0.25351 (15)	0.0304 (4)	
C23	0.13493 (13)	0.66352 (6)	0.22311 (16)	0.0362 (4)	
H23A	0.127604	0.652293	0.153767	0.054*	
H23B	0.091754	0.688497	0.214917	0.054*	
H23C	0.111248	0.642914	0.263357	0.054*	
C24	0.25876 (14)	0.70886 (6)	0.39819 (14)	0.0318 (4)	
H24A	0.235197	0.691853	0.445979	0.048*	
H24B	0.206543	0.730532	0.367829	0.048*	
H24C	0.326873	0.721546	0.437045	0.048*	
C25	0.29402 (13)	0.71554 (5)	0.20477 (13)	0.0277 (3)	
H25A	0.360776	0.729905	0.239720	0.042*	
H25B	0.235789	0.735455	0.186881	0.042*	
H25C	0.296706	0.702333	0.140576	0.042*	
C26	0.25739 (16)	0.59587 (7)	0.49069 (17)	0.0423 (5)	
H26A	0.218956	0.622090	0.481403	0.063*	
H26B	0.268261	0.585057	0.561185	0.063*	
H26C	0.216388	0.575950	0.438222	0.063*	
C27	0.44892 (18)	0.55442 (6)	0.51818 (19)	0.0483 (5)	
H27A	0.413118	0.532945	0.467719	0.072*	
H27B	0.444629	0.547685	0.587207	0.072*	
H27C	0.523174	0.555953	0.523061	0.072*	
C28	0.45209 (15)	0.63728 (7)	0.58702 (14)	0.0386 (4)	
H28A	0.524819	0.642292	0.589825	0.058*	
H28B	0.452689	0.623560	0.651760	0.058*	
H28C	0.414578	0.663656	0.579768	0.058*	
Fe2	1.06717 (2)	0.59728 (2)	0.73660 (2)	0.01677 (5)	
S2	0.86582 (3)	0.79761 (2)	0.67762 (3)	0.01875 (7)	
P3	1.20661 (3)	0.63524 (2)	0.82008 (3)	0.02169 (8)	
P4	1.05319 (3)	0.56362 (2)	0.87131 (3)	0.02180 (8)	
O2	1.21287 (10)	0.53829 (4)	0.69799 (10)	0.0350 (3)	
N4	0.98932 (9)	0.67679 (4)	0.78335 (9)	0.0182 (2)	
N5	0.96964 (9)	0.64174 (4)	0.72141 (9)	0.0171 (2)	
N6	0.94560 (10)	0.57373 (4)	0.62879 (9)	0.0210 (2)	
C29	0.88599 (11)	0.84938 (4)	0.71594 (12)	0.0184 (3)	
C30	0.86403 (11)	0.88323 (5)	0.64814 (13)	0.0233 (3)	
H30	0.833657	0.879685	0.574355	0.028*	
C31	0.88807 (13)	0.92220 (5)	0.69217 (14)	0.0276 (3)	
H31	0.874472	0.945738	0.647704	0.033*	
C32	0.93179 (13)	0.92747 (5)	0.80038 (15)	0.0287 (3)	
H32	0.946909	0.954560	0.828425	0.034*	
C33	0.95368 (12)	0.89386 (5)	0.86800 (13)	0.0239 (3)	
H33	0.983475	0.897741	0.941717	0.029*	
C34	0.93101 (11)	0.85410 (4)	0.82548 (12)	0.0189 (3)	
C35	0.92194 (10)	0.78126 (4)	0.80860 (11)	0.0171 (3)	
C36	0.95100 (11)	0.81457 (4)	0.87915 (11)	0.0178 (3)	
C37	0.99331 (12)	0.80680 (5)	0.98707 (12)	0.0226 (3)	
H37	1.011412	0.829011	1.035756	0.027*	
C38	1.00841 (13)	0.76631 (5)	1.02188 (12)	0.0253 (3)	
H38	1.035745	0.760779	1.095041	0.030*	
C39	0.98387 (12)	0.73344 (5)	0.95052 (12)	0.0235 (3)	
H39	0.996967	0.705941	0.976125	0.028*	
C40	0.94065 (11)	0.74017 (4)	0.84278 (11)	0.0186 (3)	
C41	0.91399 (11)	0.70474 (4)	0.76852 (11)	0.0186 (3)	
C42	0.81208 (11)	0.70174 (5)	0.69147 (12)	0.0210 (3)	
H42	0.762254	0.723579	0.679550	0.025*	
C43	0.78924 (12)	0.66576 (5)	0.63519 (11)	0.0210 (3)	
H43	0.721168	0.661503	0.584742	0.025*	
C44	0.86699 (11)	0.63537 (4)	0.65263 (11)	0.0189 (3)	
C45	0.85334 (12)	0.59607 (4)	0.60339 (11)	0.0210 (3)	
C46	0.75592 (14)	0.57940 (5)	0.53921 (13)	0.0284 (3)	
H46	0.693186	0.595298	0.523872	0.034*	
C47	0.75179 (15)	0.54039 (5)	0.49910 (13)	0.0329 (4)	
H47	0.686507	0.528614	0.457233	0.039*	
C48	0.84680 (15)	0.51813 (5)	0.52147 (13)	0.0316 (4)	
H48	0.846718	0.491330	0.492899	0.038*	
C49	0.93895 (14)	0.53533 (5)	0.58444 (12)	0.0266 (3)	
H49	1.002109	0.519805	0.598322	0.032*	
C50	1.15461 (13)	0.56265 (5)	0.71191 (12)	0.0239 (3)	
C51	1.22180 (15)	0.68063 (6)	0.74707 (16)	0.0360 (4)	
H51A	1.225619	0.672082	0.678987	0.054*	
H51B	1.286782	0.695225	0.786874	0.054*	
H51C	1.161171	0.699030	0.736038	0.054*	
C52	1.22545 (14)	0.65821 (6)	0.94861 (13)	0.0319 (4)	
H52A	1.162822	0.674345	0.945412	0.048*	
H52B	1.287421	0.676315	0.968223	0.048*	
H52C	1.236191	0.636259	1.001044	0.048*	
C53	1.33634 (13)	0.61128 (6)	0.84742 (16)	0.0352 (4)	
H53A	1.342898	0.587641	0.894428	0.053*	
H53B	1.391603	0.631508	0.880919	0.053*	
H53C	1.343967	0.601847	0.781548	0.053*	
C54	0.96084 (15)	0.52024 (5)	0.83666 (14)	0.0322 (4)	
H54A	0.889850	0.530472	0.797736	0.048*	
H54B	0.960213	0.506353	0.900773	0.048*	
H54C	0.982970	0.500661	0.792901	0.048*	
C55	1.17149 (14)	0.53884 (6)	0.96047 (14)	0.0340 (4)	
H55A	1.202824	0.521050	0.920264	0.051*	
H55B	1.152500	0.522139	1.011815	0.051*	
H55C	1.222594	0.560041	0.997153	0.051*	
C56	1.00203 (15)	0.59247 (6)	0.95985 (14)	0.0337 (4)	
H56A	1.046068	0.616895	0.985942	0.051*	
H56B	1.003260	0.574851	1.019124	0.051*	
H56C	0.929324	0.601124	0.921955	0.051*	

### Carbonyl[3-(dibenzo[b,d]thiophen-4-yl)-6-(pyridin-2-yl-κN)pyridazine-κN1]bis(trimethylphosphane)iron(0) Atomic displacement parameters (Å^2^)

**Table:**

	*U*^11^	*U*^22^	*U*^33^	*U*^12^	*U*^13^	*U*^23^
Fe1	0.01493 (10)	0.02206 (11)	0.02349 (11)	−0.00260 (7)	0.00833 (8)	−0.00272 (8)
S1	0.0319 (2)	0.02150 (18)	0.02929 (19)	−0.00082 (15)	0.01718 (17)	−0.00173 (15)
P1	0.01522 (17)	0.0289 (2)	0.02408 (19)	0.00079 (14)	0.00856 (15)	−0.00361 (15)
P2	0.02358 (19)	0.0303 (2)	0.0277 (2)	−0.00653 (16)	0.01035 (17)	0.00340 (17)
O1	0.0299 (7)	0.0471 (8)	0.0618 (10)	−0.0133 (6)	0.0081 (7)	−0.0225 (7)
N1	0.0170 (6)	0.0216 (6)	0.0219 (6)	−0.0009 (4)	0.0077 (5)	−0.0015 (5)
N2	0.0164 (5)	0.0190 (6)	0.0207 (6)	0.0004 (4)	0.0080 (5)	0.0000 (5)
N3	0.0206 (6)	0.0210 (6)	0.0248 (6)	−0.0009 (5)	0.0092 (5)	−0.0003 (5)
C1	0.0201 (7)	0.0223 (7)	0.0281 (8)	−0.0001 (6)	0.0038 (6)	−0.0017 (6)
C2	0.0310 (9)	0.0289 (8)	0.0408 (10)	−0.0016 (7)	0.0145 (8)	0.0022 (7)
C3	0.0304 (9)	0.0254 (8)	0.0521 (11)	−0.0039 (7)	0.0103 (8)	0.0042 (8)
C4	0.0243 (8)	0.0220 (8)	0.0467 (10)	−0.0007 (6)	−0.0001 (8)	−0.0057 (7)
C5	0.0225 (7)	0.0262 (8)	0.0339 (9)	0.0006 (6)	0.0007 (7)	−0.0079 (7)
C6	0.0158 (6)	0.0250 (7)	0.0251 (7)	−0.0005 (5)	−0.0005 (6)	−0.0051 (6)
C7	0.0147 (6)	0.0261 (7)	0.0197 (7)	−0.0007 (5)	0.0042 (5)	−0.0037 (6)
C8	0.0165 (6)	0.0249 (7)	0.0216 (7)	−0.0005 (5)	0.0012 (6)	−0.0060 (6)
C9	0.0275 (8)	0.0317 (8)	0.0261 (8)	−0.0016 (6)	0.0087 (7)	−0.0127 (7)
C10	0.0305 (8)	0.0396 (9)	0.0246 (8)	−0.0060 (7)	0.0133 (7)	−0.0093 (7)
C11	0.0235 (7)	0.0314 (8)	0.0222 (7)	−0.0048 (6)	0.0083 (6)	−0.0047 (6)
C12	0.0154 (6)	0.0251 (7)	0.0197 (6)	−0.0007 (5)	0.0045 (5)	−0.0030 (6)
C13	0.0173 (6)	0.0211 (7)	0.0189 (6)	0.0008 (5)	0.0058 (5)	0.0010 (5)
C14	0.0163 (6)	0.0223 (7)	0.0281 (7)	−0.0023 (5)	0.0082 (6)	0.0013 (6)
C15	0.0154 (6)	0.0235 (7)	0.0280 (7)	0.0023 (5)	0.0101 (6)	0.0027 (6)
C16	0.0149 (6)	0.0209 (7)	0.0191 (6)	0.0021 (5)	0.0068 (5)	0.0035 (5)
C17	0.0185 (6)	0.0200 (7)	0.0202 (7)	0.0017 (5)	0.0074 (6)	0.0031 (5)
C18	0.0216 (7)	0.0228 (7)	0.0293 (8)	0.0040 (6)	0.0112 (6)	0.0034 (6)
C19	0.0290 (8)	0.0255 (8)	0.0310 (8)	0.0070 (6)	0.0131 (7)	0.0001 (6)
C20	0.0332 (9)	0.0218 (8)	0.0358 (9)	−0.0002 (6)	0.0121 (7)	−0.0050 (7)
C21	0.0286 (8)	0.0224 (8)	0.0371 (9)	−0.0044 (6)	0.0126 (7)	−0.0048 (7)
C22	0.0233 (8)	0.0328 (9)	0.0357 (9)	−0.0017 (6)	0.0107 (7)	−0.0079 (7)
C23	0.0161 (7)	0.0457 (11)	0.0442 (10)	0.0005 (7)	0.0069 (7)	−0.0062 (8)
C24	0.0289 (8)	0.0390 (10)	0.0320 (9)	0.0040 (7)	0.0164 (7)	−0.0068 (7)
C25	0.0270 (8)	0.0296 (8)	0.0266 (8)	0.0050 (6)	0.0092 (7)	0.0002 (6)
C26	0.0353 (10)	0.0569 (13)	0.0407 (10)	−0.0166 (9)	0.0206 (9)	0.0025 (9)
C27	0.0514 (12)	0.0376 (11)	0.0561 (13)	0.0040 (9)	0.0186 (11)	0.0183 (10)
C28	0.0373 (10)	0.0510 (11)	0.0256 (8)	−0.0112 (8)	0.0081 (8)	0.0012 (8)
Fe2	0.02222 (10)	0.01360 (10)	0.01504 (9)	0.00217 (7)	0.00699 (8)	−0.00007 (7)
S2	0.01849 (16)	0.01793 (16)	0.01829 (15)	0.00061 (12)	0.00416 (13)	−0.00110 (13)
P3	0.02033 (18)	0.02093 (18)	0.02220 (18)	0.00205 (14)	0.00503 (15)	−0.00170 (14)
P4	0.02679 (19)	0.02252 (19)	0.01832 (17)	0.00484 (15)	0.01053 (15)	0.00397 (14)
O2	0.0410 (7)	0.0282 (6)	0.0391 (7)	0.0111 (5)	0.0177 (6)	−0.0055 (5)
N4	0.0203 (6)	0.0151 (5)	0.0181 (5)	0.0013 (4)	0.0049 (5)	−0.0019 (4)
N5	0.0208 (6)	0.0141 (5)	0.0152 (5)	−0.0003 (4)	0.0045 (5)	−0.0007 (4)
N6	0.0314 (7)	0.0159 (6)	0.0159 (5)	−0.0019 (5)	0.0084 (5)	−0.0018 (4)
C29	0.0134 (6)	0.0176 (6)	0.0248 (7)	0.0004 (5)	0.0074 (6)	−0.0010 (5)
C30	0.0179 (7)	0.0235 (7)	0.0297 (8)	−0.0002 (5)	0.0094 (6)	0.0032 (6)
C31	0.0258 (8)	0.0204 (7)	0.0396 (9)	−0.0014 (6)	0.0149 (7)	0.0050 (7)
C32	0.0285 (8)	0.0179 (7)	0.0431 (10)	−0.0048 (6)	0.0165 (7)	−0.0052 (7)
C33	0.0218 (7)	0.0217 (7)	0.0305 (8)	−0.0037 (6)	0.0116 (6)	−0.0070 (6)
C34	0.0137 (6)	0.0193 (7)	0.0259 (7)	0.0000 (5)	0.0094 (6)	−0.0029 (6)
C35	0.0139 (6)	0.0194 (7)	0.0176 (6)	0.0017 (5)	0.0049 (5)	−0.0009 (5)
C36	0.0134 (6)	0.0194 (7)	0.0215 (7)	0.0014 (5)	0.0071 (5)	−0.0038 (5)
C37	0.0217 (7)	0.0249 (7)	0.0212 (7)	0.0011 (6)	0.0072 (6)	−0.0064 (6)
C38	0.0290 (8)	0.0283 (8)	0.0170 (7)	0.0040 (6)	0.0057 (6)	−0.0008 (6)
C39	0.0257 (7)	0.0219 (7)	0.0212 (7)	0.0047 (6)	0.0058 (6)	0.0012 (6)
C40	0.0163 (6)	0.0187 (7)	0.0203 (6)	0.0026 (5)	0.0056 (5)	−0.0024 (5)
C41	0.0205 (7)	0.0164 (6)	0.0189 (6)	0.0008 (5)	0.0067 (6)	0.0009 (5)
C42	0.0186 (7)	0.0193 (7)	0.0236 (7)	0.0026 (5)	0.0053 (6)	0.0023 (6)
C43	0.0192 (7)	0.0215 (7)	0.0190 (6)	−0.0019 (5)	0.0021 (6)	0.0029 (5)
C44	0.0223 (7)	0.0172 (7)	0.0159 (6)	−0.0018 (5)	0.0046 (6)	0.0015 (5)
C45	0.0271 (7)	0.0191 (7)	0.0155 (6)	−0.0032 (5)	0.0052 (6)	0.0010 (5)
C46	0.0314 (8)	0.0254 (8)	0.0223 (7)	−0.0061 (6)	0.0011 (7)	0.0001 (6)
C47	0.0406 (10)	0.0274 (8)	0.0247 (8)	−0.0136 (7)	0.0032 (7)	−0.0032 (6)
C48	0.0516 (11)	0.0201 (7)	0.0234 (7)	−0.0100 (7)	0.0130 (8)	−0.0061 (6)
C49	0.0425 (9)	0.0177 (7)	0.0221 (7)	−0.0029 (6)	0.0143 (7)	−0.0026 (6)
C50	0.0320 (8)	0.0200 (7)	0.0209 (7)	0.0008 (6)	0.0107 (6)	0.0002 (6)
C51	0.0334 (9)	0.0299 (9)	0.0422 (10)	−0.0084 (7)	0.0093 (8)	0.0046 (8)
C52	0.0268 (8)	0.0351 (9)	0.0280 (8)	0.0031 (7)	0.0015 (7)	−0.0101 (7)
C53	0.0226 (8)	0.0390 (10)	0.0423 (10)	0.0048 (7)	0.0085 (7)	−0.0075 (8)
C54	0.0405 (10)	0.0286 (8)	0.0325 (9)	−0.0024 (7)	0.0188 (8)	0.0058 (7)
C55	0.0349 (9)	0.0385 (10)	0.0288 (8)	0.0096 (7)	0.0110 (7)	0.0135 (7)
C56	0.0387 (9)	0.0427 (10)	0.0244 (8)	0.0059 (8)	0.0168 (7)	−0.0033 (7)

### Carbonyl[3-(dibenzo[b,d]thiophen-4-yl)-6-(pyridin-2-yl-κN)pyridazine-κN1]bis(trimethylphosphane)iron(0) Geometric parameters (Å, º)

**Table:**

Fe1—P1	2.2144 (4)	Fe2—P3	2.2087 (4)
Fe1—P2	2.1847 (5)	Fe2—P4	2.1862 (4)
Fe1—N2	1.9054 (12)	Fe2—N5	1.9117 (12)
Fe1—N3	1.9464 (13)	Fe2—N6	1.9434 (13)
Fe1—C22	1.7426 (17)	Fe2—C50	1.7371 (16)
S1—C1	1.7538 (16)	S2—C29	1.7486 (15)
S1—C7	1.7547 (16)	S2—C35	1.7582 (14)
P1—C23	1.8261 (17)	P3—C51	1.8207 (18)
P1—C24	1.8382 (17)	P3—C52	1.8294 (17)
P1—C25	1.8254 (17)	P3—C53	1.8304 (17)
P2—C26	1.8258 (19)	P4—C54	1.8284 (18)
P2—C27	1.830 (2)	P4—C55	1.8271 (17)
P2—C28	1.8312 (19)	P4—C56	1.8272 (17)
O1—C22	1.169 (2)	O2—C50	1.1713 (19)
N1—N2	1.3736 (17)	N4—N5	1.3826 (16)
N1—C13	1.3224 (19)	N4—C41	1.3228 (18)
N2—C16	1.3954 (18)	N5—C44	1.3964 (18)
N3—C17	1.3830 (19)	N6—C45	1.377 (2)
N3—C21	1.375 (2)	N6—C49	1.3704 (19)
C1—C2	1.397 (2)	C29—C30	1.396 (2)
C1—C6	1.401 (2)	C29—C34	1.408 (2)
C2—H2	0.9500	C30—H30	0.9500
C2—C3	1.386 (3)	C30—C31	1.386 (2)
C3—H3	0.9500	C31—H31	0.9500
C3—C4	1.395 (3)	C31—C32	1.393 (3)
C4—H4	0.9500	C32—H32	0.9500
C4—C5	1.382 (3)	C32—C33	1.388 (2)
C5—H5	0.9500	C33—H33	0.9500
C5—C6	1.401 (2)	C33—C34	1.401 (2)
C6—C8	1.449 (2)	C34—C36	1.451 (2)
C7—C8	1.410 (2)	C35—C36	1.406 (2)
C7—C12	1.404 (2)	C35—C40	1.403 (2)
C8—C9	1.397 (2)	C36—C37	1.400 (2)
C9—H9	0.9500	C37—H37	0.9500
C9—C10	1.375 (3)	C37—C38	1.385 (2)
C10—H10	0.9500	C38—H38	0.9500
C10—C11	1.397 (2)	C38—C39	1.400 (2)
C11—H11	0.9500	C39—H39	0.9500
C11—C12	1.394 (2)	C39—C40	1.393 (2)
C12—C13	1.489 (2)	C40—C41	1.487 (2)
C13—C14	1.420 (2)	C41—C42	1.419 (2)
C14—H14	0.9500	C42—H42	0.9500
C14—C15	1.368 (2)	C42—C43	1.369 (2)
C15—H15	0.9500	C43—H43	0.9500
C15—C16	1.401 (2)	C43—C44	1.397 (2)
C16—C17	1.411 (2)	C44—C45	1.419 (2)
C17—C18	1.412 (2)	C45—C46	1.413 (2)
C18—H18	0.9500	C46—H46	0.9500
C18—C19	1.365 (2)	C46—C47	1.369 (2)
C19—H19	0.9500	C47—H47	0.9500
C19—C20	1.408 (2)	C47—C48	1.409 (3)
C20—H20	0.9500	C48—H48	0.9500
C20—C21	1.368 (2)	C48—C49	1.364 (2)
C21—H21	0.9500	C49—H49	0.9500
C23—H23A	0.9800	C51—H51A	0.9800
C23—H23B	0.9800	C51—H51B	0.9800
C23—H23C	0.9800	C51—H51C	0.9800
C24—H24A	0.9800	C52—H52A	0.9800
C24—H24B	0.9800	C52—H52B	0.9800
C24—H24C	0.9800	C52—H52C	0.9800
C25—H25A	0.9800	C53—H53A	0.9800
C25—H25B	0.9800	C53—H53B	0.9800
C25—H25C	0.9800	C53—H53C	0.9800
C26—H26A	0.9800	C54—H54A	0.9800
C26—H26B	0.9800	C54—H54B	0.9800
C26—H26C	0.9800	C54—H54C	0.9800
C27—H27A	0.9800	C55—H55A	0.9800
C27—H27B	0.9800	C55—H55B	0.9800
C27—H27C	0.9800	C55—H55C	0.9800
C28—H28A	0.9800	C56—H56A	0.9800
C28—H28B	0.9800	C56—H56B	0.9800
C28—H28C	0.9800	C56—H56C	0.9800
			
H9···C17^i^	2.676	H23C···C34^iii^	2.824
H15···C34^ii^	2.704	H25C···C52^v^	2.834
H2···C43^ii^	2.730	H49^vi^···H32^iii^	2.336
C8···H53B^iii^	2.755	C6···H30	2.841
H15···C36^ii^	2.770	H23B···S2^iii^	2.943
S1···C42^ii^	3.389	H5···C19^i^	2.843
O2···H48^iv^	2.616	H19···O2^iv^	2.664
H18···C32^ii^	2.798		
			
P2—Fe1—P1	99.457 (18)	P4—Fe2—P3	98.499 (17)
N2—Fe1—P1	91.04 (4)	N5—Fe2—P3	94.01 (4)
N2—Fe1—P2	102.65 (4)	N5—Fe2—P4	102.48 (4)
N2—Fe1—N3	80.70 (5)	N5—Fe2—N6	80.86 (5)
N3—Fe1—P1	153.19 (4)	N6—Fe2—P3	163.19 (4)
N3—Fe1—P2	107.22 (4)	N6—Fe2—P4	98.24 (4)
C22—Fe1—P1	88.71 (6)	C50—Fe2—P3	86.79 (5)
C22—Fe1—P2	93.24 (6)	C50—Fe2—P4	95.33 (5)
C22—Fe1—N2	163.93 (7)	C50—Fe2—N5	161.83 (6)
C22—Fe1—N3	92.38 (7)	C50—Fe2—N6	93.19 (7)
C1—S1—C7	91.40 (8)	C29—S2—C35	91.07 (7)
C23—P1—Fe1	115.71 (7)	C51—P3—Fe2	113.76 (6)
C23—P1—C24	100.05 (8)	C51—P3—C52	100.55 (9)
C24—P1—Fe1	123.45 (6)	C51—P3—C53	100.26 (9)
C25—P1—Fe1	113.27 (5)	C52—P3—Fe2	122.29 (6)
C25—P1—C23	100.13 (9)	C52—P3—C53	99.38 (8)
C25—P1—C24	100.75 (8)	C53—P3—Fe2	117.08 (6)
C26—P2—Fe1	117.72 (7)	C54—P4—Fe2	114.27 (6)
C26—P2—C27	100.80 (10)	C55—P4—Fe2	117.97 (6)
C26—P2—C28	101.83 (10)	C55—P4—C54	101.40 (9)
C27—P2—Fe1	114.64 (8)	C55—P4—C56	103.19 (9)
C27—P2—C28	101.53 (11)	C56—P4—Fe2	116.71 (6)
C28—P2—Fe1	117.66 (6)	C56—P4—C54	100.79 (9)
C13—N1—N2	119.81 (12)	C41—N4—N5	119.30 (12)
N1—N2—Fe1	123.88 (9)	N4—N5—Fe2	124.66 (9)
N1—N2—C16	118.74 (12)	N4—N5—C44	118.02 (11)
C16—N2—Fe1	117.26 (10)	C44—N5—Fe2	116.70 (9)
C17—N3—Fe1	116.15 (10)	C45—N6—Fe2	116.19 (10)
C21—N3—Fe1	127.66 (11)	C49—N6—Fe2	126.80 (11)
C21—N3—C17	116.18 (13)	C49—N6—C45	116.56 (13)
C2—C1—S1	126.37 (14)	C30—C29—S2	125.48 (12)
C2—C1—C6	121.30 (15)	C30—C29—C34	121.85 (14)
C6—C1—S1	112.33 (12)	C34—C29—S2	112.64 (11)
C1—C2—H2	120.8	C29—C30—H30	121.1
C3—C2—C1	118.48 (18)	C31—C30—C29	117.71 (15)
C3—C2—H2	120.8	C31—C30—H30	121.1
C2—C3—H3	119.6	C30—C31—H31	119.4
C2—C3—C4	120.83 (17)	C30—C31—C32	121.23 (15)
C4—C3—H3	119.6	C32—C31—H31	119.4
C3—C4—H4	119.7	C31—C32—H32	119.4
C5—C4—C3	120.63 (16)	C33—C32—C31	121.17 (15)
C5—C4—H4	119.7	C33—C32—H32	119.4
C4—C5—H5	120.2	C32—C33—H33	120.6
C4—C5—C6	119.60 (17)	C32—C33—C34	118.76 (15)
C6—C5—H5	120.2	C34—C33—H33	120.6
C1—C6—C5	119.16 (16)	C29—C34—C36	111.88 (12)
C1—C6—C8	112.15 (14)	C33—C34—C29	119.27 (14)
C5—C6—C8	128.69 (16)	C33—C34—C36	128.81 (14)
C8—C7—S1	111.88 (12)	C36—C35—S2	112.35 (11)
C12—C7—S1	126.64 (11)	C40—C35—S2	125.90 (11)
C12—C7—C8	121.45 (14)	C40—C35—C36	121.71 (13)
C7—C8—C6	112.24 (14)	C35—C36—C34	111.99 (13)
C9—C8—C6	128.45 (14)	C37—C36—C34	128.48 (13)
C9—C8—C7	119.29 (15)	C37—C36—C35	119.53 (13)
C8—C9—H9	120.1	C36—C37—H37	120.4
C10—C9—C8	119.73 (15)	C38—C37—C36	119.11 (14)
C10—C9—H9	120.1	C38—C37—H37	120.4
C9—C10—H10	119.7	C37—C38—H38	119.6
C9—C10—C11	120.65 (15)	C37—C38—C39	120.78 (14)
C11—C10—H10	119.7	C39—C38—H38	119.6
C10—C11—H11	119.3	C38—C39—H39	119.3
C12—C11—C10	121.47 (16)	C40—C39—C38	121.43 (14)
C12—C11—H11	119.3	C40—C39—H39	119.3
C7—C12—C13	123.21 (13)	C35—C40—C41	122.20 (13)
C11—C12—C7	117.37 (14)	C39—C40—C35	117.30 (13)
C11—C12—C13	119.40 (14)	C39—C40—C41	120.49 (13)
N1—C13—C12	113.63 (12)	N4—C41—C40	115.29 (12)
N1—C13—C14	123.87 (14)	N4—C41—C42	124.46 (13)
C14—C13—C12	122.46 (13)	C42—C41—C40	120.19 (13)
C13—C14—H14	121.7	C41—C42—H42	121.7
C15—C14—C13	116.64 (13)	C43—C42—C41	116.58 (13)
C15—C14—H14	121.7	C43—C42—H42	121.7
C14—C15—H15	120.0	C42—C43—H43	120.3
C14—C15—C16	119.95 (13)	C42—C43—C44	119.37 (13)
C16—C15—H15	120.0	C44—C43—H43	120.3
N2—C16—C15	120.66 (13)	N5—C44—C43	121.65 (13)
N2—C16—C17	112.76 (12)	N5—C44—C45	112.81 (13)
C15—C16—C17	126.55 (13)	C43—C44—C45	125.53 (14)
N3—C17—C16	113.11 (12)	N6—C45—C44	113.00 (13)
N3—C17—C18	121.58 (14)	N6—C45—C46	121.63 (14)
C16—C17—C18	125.30 (14)	C46—C45—C44	125.28 (15)
C17—C18—H18	119.8	C45—C46—H46	119.9
C19—C18—C17	120.39 (15)	C47—C46—C45	120.11 (16)
C19—C18—H18	119.8	C47—C46—H46	119.9
C18—C19—H19	120.8	C46—C47—H47	120.8
C18—C19—C20	118.38 (15)	C46—C47—C48	118.31 (16)
C20—C19—H19	120.8	C48—C47—H47	120.8
C19—C20—H20	120.2	C47—C48—H48	120.2
C21—C20—C19	119.53 (15)	C49—C48—C47	119.56 (15)
C21—C20—H20	120.2	C49—C48—H48	120.2
N3—C21—H21	118.1	N6—C49—H49	118.1
C20—C21—N3	123.81 (15)	C48—C49—N6	123.73 (16)
C20—C21—H21	118.1	C48—C49—H49	118.1
O1—C22—Fe1	177.53 (18)	O2—C50—Fe2	177.43 (14)
P1—C23—H23A	109.5	P3—C51—H51A	109.5
P1—C23—H23B	109.5	P3—C51—H51B	109.5
P1—C23—H23C	109.5	P3—C51—H51C	109.5
H23A—C23—H23B	109.5	H51A—C51—H51B	109.5
H23A—C23—H23C	109.5	H51A—C51—H51C	109.5
H23B—C23—H23C	109.5	H51B—C51—H51C	109.5
P1—C24—H24A	109.5	P3—C52—H52A	109.5
P1—C24—H24B	109.5	P3—C52—H52B	109.5
P1—C24—H24C	109.5	P3—C52—H52C	109.5
H24A—C24—H24B	109.5	H52A—C52—H52B	109.5
H24A—C24—H24C	109.5	H52A—C52—H52C	109.5
H24B—C24—H24C	109.5	H52B—C52—H52C	109.5
P1—C25—H25A	109.5	P3—C53—H53A	109.5
P1—C25—H25B	109.5	P3—C53—H53B	109.5
P1—C25—H25C	109.5	P3—C53—H53C	109.5
H25A—C25—H25B	109.5	H53A—C53—H53B	109.5
H25A—C25—H25C	109.5	H53A—C53—H53C	109.5
H25B—C25—H25C	109.5	H53B—C53—H53C	109.5
P2—C26—H26A	109.5	P4—C54—H54A	109.5
P2—C26—H26B	109.5	P4—C54—H54B	109.5
P2—C26—H26C	109.5	P4—C54—H54C	109.5
H26A—C26—H26B	109.5	H54A—C54—H54B	109.5
H26A—C26—H26C	109.5	H54A—C54—H54C	109.5
H26B—C26—H26C	109.5	H54B—C54—H54C	109.5
P2—C27—H27A	109.5	P4—C55—H55A	109.5
P2—C27—H27B	109.5	P4—C55—H55B	109.5
P2—C27—H27C	109.5	P4—C55—H55C	109.5
H27A—C27—H27B	109.5	H55A—C55—H55B	109.5
H27A—C27—H27C	109.5	H55A—C55—H55C	109.5
H27B—C27—H27C	109.5	H55B—C55—H55C	109.5
P2—C28—H28A	109.5	P4—C56—H56A	109.5
P2—C28—H28B	109.5	P4—C56—H56B	109.5
P2—C28—H28C	109.5	P4—C56—H56C	109.5
H28A—C28—H28B	109.5	H56A—C56—H56B	109.5
H28A—C28—H28C	109.5	H56A—C56—H56C	109.5
H28B—C28—H28C	109.5	H56B—C56—H56C	109.5
			
Fe1—N2—C16—C15	177.72 (11)	Fe2—N5—C44—C43	−179.81 (11)
Fe1—N2—C16—C17	−0.56 (16)	Fe2—N5—C44—C45	−1.00 (16)
Fe1—N3—C17—C16	1.61 (16)	Fe2—N6—C45—C44	7.11 (16)
Fe1—N3—C17—C18	−177.05 (11)	Fe2—N6—C45—C46	−169.65 (12)
Fe1—N3—C21—C20	178.79 (14)	Fe2—N6—C49—C48	169.22 (13)
S1—C1—C2—C3	179.57 (14)	S2—C29—C30—C31	−178.05 (12)
S1—C1—C6—C5	−179.88 (12)	S2—C29—C34—C33	178.83 (11)
S1—C1—C6—C8	0.40 (16)	S2—C29—C34—C36	0.92 (15)
S1—C7—C8—C6	0.83 (16)	S2—C35—C36—C34	−2.24 (15)
S1—C7—C8—C9	179.46 (12)	S2—C35—C36—C37	177.62 (11)
S1—C7—C12—C11	−179.76 (12)	S2—C35—C40—C39	−178.63 (11)
S1—C7—C12—C13	−1.4 (2)	S2—C35—C40—C41	0.1 (2)
N1—N2—C16—C15	−6.2 (2)	N4—N5—C44—C43	8.8 (2)
N1—N2—C16—C17	175.51 (12)	N4—N5—C44—C45	−172.39 (12)
N1—C13—C14—C15	−3.2 (2)	N4—C41—C42—C43	5.3 (2)
N2—N1—C13—C12	−178.43 (12)	N5—N4—C41—C40	176.74 (12)
N2—N1—C13—C14	−0.8 (2)	N5—N4—C41—C42	−0.6 (2)
N2—C16—C17—N3	−0.68 (18)	N5—C44—C45—N6	−3.89 (18)
N2—C16—C17—C18	177.92 (14)	N5—C44—C45—C46	172.73 (14)
N3—C17—C18—C19	−2.7 (2)	N6—C45—C46—C47	−1.1 (2)
C1—S1—C7—C8	−0.51 (12)	C29—S2—C35—C36	2.34 (11)
C1—S1—C7—C12	177.29 (14)	C29—S2—C35—C40	−175.61 (13)
C1—C2—C3—C4	0.8 (3)	C29—C30—C31—C32	−0.5 (2)
C1—C6—C8—C7	−0.79 (18)	C29—C34—C36—C35	0.85 (17)
C1—C6—C8—C9	−179.26 (15)	C29—C34—C36—C37	−178.99 (14)
C2—C1—C6—C5	0.3 (2)	C30—C29—C34—C33	0.6 (2)
C2—C1—C6—C8	−179.40 (15)	C30—C29—C34—C36	−177.34 (13)
C2—C3—C4—C5	−0.7 (3)	C30—C31—C32—C33	0.5 (2)
C3—C4—C5—C6	0.3 (2)	C31—C32—C33—C34	0.0 (2)
C4—C5—C6—C1	−0.1 (2)	C32—C33—C34—C29	−0.6 (2)
C4—C5—C6—C8	179.53 (15)	C32—C33—C34—C36	176.94 (14)
C5—C6—C8—C7	179.52 (15)	C33—C34—C36—C35	−176.81 (14)
C5—C6—C8—C9	1.0 (3)	C33—C34—C36—C37	3.3 (2)
C6—C1—C2—C3	−0.7 (2)	C34—C29—C30—C31	0.0 (2)
C6—C8—C9—C10	178.74 (15)	C34—C36—C37—C38	−178.36 (14)
C7—S1—C1—C2	179.85 (15)	C35—S2—C29—C30	176.34 (13)
C7—S1—C1—C6	0.06 (12)	C35—S2—C29—C34	−1.85 (11)
C7—C8—C9—C10	0.4 (2)	C35—C36—C37—C38	1.8 (2)
C7—C12—C13—N1	−134.90 (15)	C35—C40—C41—N4	132.23 (14)
C7—C12—C13—C14	47.4 (2)	C35—C40—C41—C42	−50.3 (2)
C8—C7—C12—C11	−2.2 (2)	C36—C35—C40—C39	3.6 (2)
C8—C7—C12—C13	176.22 (13)	C36—C35—C40—C41	−177.64 (13)
C8—C9—C10—C11	−1.5 (3)	C36—C37—C38—C39	1.3 (2)
C9—C10—C11—C12	0.9 (3)	C37—C38—C39—C40	−2.0 (2)
C10—C11—C12—C7	1.0 (2)	C38—C39—C40—C35	−0.5 (2)
C10—C11—C12—C13	−177.47 (14)	C38—C39—C40—C41	−179.24 (14)
C11—C12—C13—N1	43.44 (19)	C39—C40—C41—N4	−49.0 (2)
C11—C12—C13—C14	−134.22 (16)	C39—C40—C41—C42	128.39 (16)
C12—C7—C8—C6	−177.10 (13)	C40—C35—C36—C34	175.81 (13)
C12—C7—C8—C9	1.5 (2)	C40—C35—C36—C37	−4.3 (2)
C12—C13—C14—C15	174.25 (14)	C40—C41—C42—C43	−171.86 (14)
C13—N1—N2—Fe1	−178.75 (10)	C41—N4—N5—Fe2	−177.03 (10)
C13—N1—N2—C16	5.46 (19)	C41—N4—N5—C44	−6.40 (19)
C13—C14—C15—C16	2.3 (2)	C41—C42—C43—C44	−2.8 (2)
C14—C15—C16—N2	2.2 (2)	C42—C43—C44—N5	−4.1 (2)
C14—C15—C16—C17	−179.76 (15)	C42—C43—C44—C45	177.30 (14)
C15—C16—C17—N3	−178.83 (14)	C43—C44—C45—N6	174.86 (14)
C15—C16—C17—C18	−0.2 (2)	C43—C44—C45—C46	−8.5 (2)
C16—C17—C18—C19	178.76 (15)	C44—C45—C46—C47	−177.45 (15)
C17—N3—C21—C20	−2.7 (2)	C45—N6—C49—C48	−2.7 (2)
C17—C18—C19—C20	−0.6 (2)	C45—C46—C47—C48	−1.5 (3)
C18—C19—C20—C21	2.1 (3)	C46—C47—C48—C49	2.0 (3)
C19—C20—C21—N3	−0.5 (3)	C47—C48—C49—N6	0.2 (3)
C21—N3—C17—C16	−177.08 (14)	C49—N6—C45—C44	179.90 (13)
C21—N3—C17—C18	4.3 (2)	C49—N6—C45—C46	3.1 (2)

Symmetry codes: (i) *x*, −*y*+3/2, *z*+1/2; (ii) *x*, −*y*+3/2, *z*−1/2; (iii) *x*−1, −*y*+3/2, *z*−1/2; (iv) −*x*+2, −*y*+1, −*z*+1; (v) *x*−1, *y*, *z*−1; (vi) −*x*+1, −*y*+1, −*z*+1.
